# Correspondence

**Published:** 1873-01

**Authors:** T. J. Heard

**Affiliations:** Galveston


### CORRESPONDENCE.

GALVESTON, December 19, 1872.

*Editors Southern Medical Companion:*

The horse distemper is well-nigh universal in this city, and has been for the last month. Oxen have, in a good measure, taken the place of horses. I feel satisfied, from what I have observed of late, that the disease of which the horses have been and are now suffering is due to some broadcast atmospheric influence, which will, before Spring, result in much suffering and death to the human family; and I think it probable that diphtheria will prevail to a greater extent than it has for years. I have seen a few cases of late, and as I have every reason to be pleased with my treatment, I will in outline give it to you.

The leading object with me is to saturate the system with quinine as soon as possible. My mode of administration has been as follows: For a child three years old I give the following—

℞.—Sulph. quinine, grs. x; pulv. sacch. alba., ʒ ij. Mix. Make powders No. xx. One to be taken dry on the tongue every half-hour.

In five minutes after the quinine has been placed dry on the tongue, I give one drop of nitric acid (not diluted) in a wineglass of sweetened water. In the meantime, the neck should be well rubbed with camphorated oil; over this, cotton wool should be carefully laid, and be well secured with oil silk; this should be renewed every second or third hour. In the meantime, hot foot-baths may be used, and the patient allowed as much pleasant dilutent drinks as is wished. The patient should be warmly clad. Under this treatment, as a rule, the fever which usually attends this disease in its early stage will soon pass off. Free perspiration will be soon induced, and the quinine, with acid as a solvent, will sustain a soft, velvety condition of the skin until every vestige of the fever has left. As a rule, in six hours after this plan of treatment has been instituted, the fever will have left, and the sick will ask for food, and most of the complaint of the throat will have ceased. Even at this early stage of the disease, the diphtheritic exudation will be found exfoliating, and very often the tonsils, pharynx, etc., will be found free of it in forty-eight hours.

I remarked in the foregoing that the quinine and nitric acid should be given every half hour, but I wish to be understood that this plan should not be kept up any longer than to carry it to the point of bringing about full saturation of the system with quinine. When free perspiration has been induced, the quinine may be given at longer periods; but I am satisfied that the quinine and the acid should be continued at longer and longer intervals, as the patient improves, until every evidence of the diphtheritic exudation has sloughed off. Then, and not until then, do I use the chlorate of potassa, and a rather favorite formula with me is the following:

℞.—Pulv. chlorate potassa, ʒj; tr. ferri. chloridi, ʒj; chloride quinine, ʒss; tr. columba, ʒiv; hydrochloric acid, ʒss; aq. font., ℥iss; syr. orange peel, ℥ij: mix. Take one teaspoonful in a tablespoonful of water, every third to sixth hour.

The sick should have plenty of air, but should be well protected from cold. The diet should be very nutritious, and should be given in full quantities whenever the patient desires it. I use no caustics to the throat, nor do I purge. So the bowels are easy, is all that is necessary, and if they have to be moved by artificial means, I prefer an enema, and if this will not answer the purpose, castor oil and oil turpentine will do well.

The above thoughts have been hastily thrown together. If you think the paper worthy of a place in your journal, you may put it in. Your friend, etc., T. J. HEARD.

---

PUNCTURE OF HERNIAL SAC.—Attention has lately been attracted to a method of treating strangulated hernia by puncturing the sac with a fine needle and evacuating a portion of the contents, after which reduction is easily accomplished. There is no escape of air or liquid into the abdomen, and the puncture of the intestine is found to close immediately. The same treatment is habitually resorted to by some practitioners in abdominal tympanites, and also in distension of the bladder from urine when the catheter cannot be passed. A discussion on the subject which took place in the French Academy is minuted in the Cincinnati Clinic for January 6, 1872.

